# Supplementary material for: Measurement Accuracy and Repeatability of RECIST-Defined Pulmonary Lesions and Lymph Nodes in Ultra-Low-Dose CT Based on Deep Learning Image Reconstruction
Source: Cancers (Basel). 2022 Oct 13;14(20):5016. doi: 10.3390/cancers14205016 (PMC9599467; doi:10.3390/cancers14205016)
Supplement: Supplementary file 1 [file cancers-14-05016-s001.zip › cancers-1951175-supplementary.pdf]

Supple

Measurement accuracy and repeatability of RECIST-defined pulmonary lesions and lymph nodes in ultra-low-dose CT based on deep learning image reconstruction

Keke Zhao[1], Beibei Jiang[1], Shuai Zhang[2], Lu Zhang[1], Lin Zhang[1], Yan Feng[1], Jianying Li[2], Yaping Zhang[1], and Xueqian Xie[1]

Response Evaluation Criteria in Solid Tumors (RECIST) (https://recist.eortc.org/) provides a simple and practical method to evaluate the activity and efficacy of new cancer treatments in solid tumors, using validated and consistent criteria to evaluate the changes in tumor burden. RECIST aims to objectively assess changes in the size of solid tumors of adult and pediatric oncology clinical trials and has been widely adopted by various groups or institutions around the world. It was firstly released in 2000 based on the World Health Organization (WHO) guidelines and updated in 2009 (RECIST 1.1). It is anticipated that RECIST will be used effectively in all trials, where an objective response is the primary study endpoint, as all measures of treatment effect are based on the assessment of anatomical tumor load and its size change [1-3].

According to RECIST, the pulmonary lesions and lymph nodes can be divided into measurable and nonmeasurable categories (Table S1). For measurable tumor lesions, the long diameter should not be less than 10 mm. For measurable lymph nodes, the short diameter should not be less than 15 mm. For nonmeasurable tumor lesions, the long diameter should be less than 10 mm. For nonmeasurable lymph nodes, the short diameter should not be less than 10 mm and not greater than 15 mm. Other lesions, such as pericardial effusion and inflammatory diseases, are regarded as truly nonmeasurable [4]. When lesions merge to form an aggregate, the maximum diameter of the aggregated lesion can be measured.

The frequency of lesion evaluation depends on disease progression, organs, or any other practical issues such as patient willingness or cost. It is recommended that the follow-up be conducted every 6 to 8 weeks during the Phase II studies [1]. Therefore, repeated CT scanning is needed [5]. By calculating the change range of the measured lesion diameters over the baseline value or the optimal value, the tumor treatment response can be obtained. The tumor response includes complete response (CR), partial response (PR) and stable disease (SD), and progressive disease (PD).

Table S1. Classification of RECIST-defined lesions

**RECIST-defined lesions**	**Size range**
Measurable target lesions	
Pulmonary target lesions	≥ 10 mm
Lymph nodes	≥ 15 mm
Nonmeasurable lesions	
Pulmonary lesions	< 10 mm
Lymph nodes	10 ~ 15mm
Non-pathological lymph nodes	< 10 mm

Note: For measurable or nonmeasurable pulmonary lesions, the long diameter of the lesion should be recorded; for measurable or nonmeasurable lymph nodes, the short diameter of the lesion should be recorded. The thickness of CT images for measuring the above diameters should be ≤ 5 mm.

Figure S1. Model training diagram of DLIR algorithm

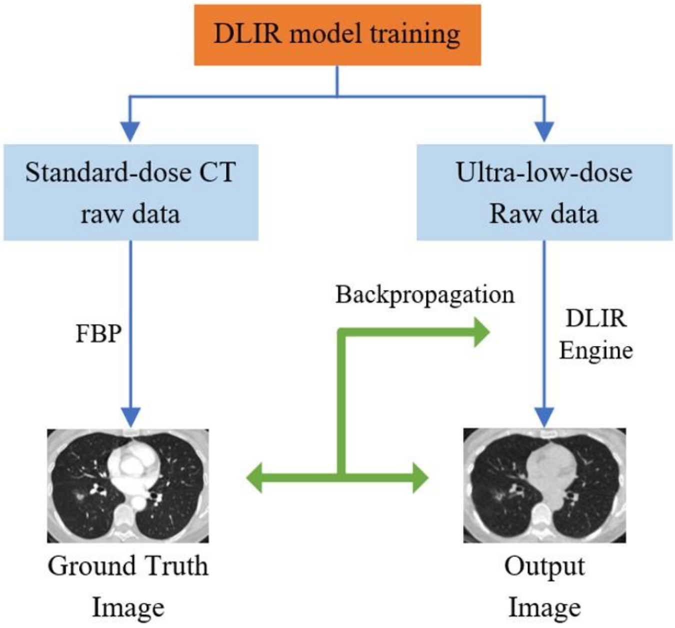

Note. In the training, the output images are generated from the raw data of ultra-low-dose CT through the DLIR engine. The output images are compared with the ground truth images of the same object generated from the raw data of standard-dose CT based on the conventional FBP algorithm. The millions of parameters in the deep neural network are adjusted by minimizing the differences between the two sets of images. More details are available at https://www.gehealthcare.com/products/truefidelity.

References:

1. Schwartz, L.H.; Litière, S.; de Vries, E.; Ford, R.; Gwyther, S.; Mandrekar, S.; Shankar, L.; Bogaerts, J.; Chen, A.; Dancey, J.; et al. RECIST 1.1-Update and clarification: From the RECIST committee. *European journal of cancer (Oxford, England : 1990)* **2016**, *62*, 132-137, doi:10.1016/j.ejca.2016.03.081.
2. Nishino, M.; Jackman, D.M.; Hatabu, H.; Yeap, B.Y.; Cioffredi, L.A.; Yap, J.T.; Jänne, P.A.; Johnson, B.E.; Van den Abbeele, A.D. New Response Evaluation Criteria in Solid Tumors (RECIST) guidelines for advanced non-small cell lung cancer: comparison with original RECIST and impact on assessment of tumor response to targeted therapy. *AJR. American journal of roentgenology* **2010**, *195*, W221-228, doi:10.2214/ajr.09.3928.
3. Litière, S.; Collette, S.; de Vries, E.G.; Seymour, L.; Bogaerts, J. RECIST - learning from the past to build the future. *Nature reviews. Clinical oncology* **2017**, *14*, 187-192, doi:10.1038/nrclinonc.2016.195.
4. Eisenhauer, E.A.; Therasse, P.; Bogaerts, J.; Schwartz, L.H.; Sargent, D.; Ford, R.; Dancey, J.; Arbuck, S.; Gwyther, S.; Mooney, M.; et al. New response evaluation criteria in solid tumours: revised RECIST guideline (version 1.1). *European journal of cancer (Oxford, England : 1990)* **2009**, *45*, 228-247, doi:10.1016/j.ejca.2008.10.026.
5. Therasse, P.; Arbuck, S.G.; Eisenhauer, E.A.; Wanders, J.; Kaplan, R.S.; Rubinstein, L.; Verweij, J.; Van Glabbeke, M.; van Oosterom, A.T.; Christian, M.C.; et al. New guidelines to evaluate the response to treatment in solid tumors. European Organization for Research and Treatment of Cancer, National Cancer Institute of the United States, National Cancer Institute of Canada. *Journal of the National Cancer Institute* **2000**, *92*, 205-216, doi:10.1093/jnci/92.3.205.
